# Sorafenib versus sunitinib as first-line treatment agents in Chinese patients with metastatic renal cell carcinoma: the largest multicenter retrospective analysis of survival and prognostic factors

**DOI:** 10.1186/s12885-016-3016-4

**Published:** 2017-01-05

**Authors:** Hai-Liang Zhang, Xi-Nan Sheng, Xue-Song Li, Hong-Kai Wang, Zhi-Hong Chi, Zhi-Song He, Ding-Wei Ye, Jun Guo

**Affiliations:** 1 Department of Urology, Fudan University Shanghai Cancer Center, Shanghai, 200032 People’s Republic of China; 2 Department of Oncology, Shanghai Medical College, Fudan University, Shanghai, 200032 People’s Republic of China; 3 Key Laboratory of Carcinogenesis and Translational Research (Ministry of Education/Beijing), Department of Renal Cancer and Melanoma, Peking University Cancer Hospital & Institute, Beijing, People’s Republic of China; 4 Department of Urology, Peking University First Hospital, Institute of Urology, National Urological Cancer Center, Peking University, Beijing, People’s Republic of China

**Keywords:** Metastatic renal cell carcinoma, Sorafenib, Sunitinib, Prognosis, Survival

## Abstract

**Background:**

To compare the efficacy of sorafenib and sunitinib with regard to overall survival (OS) and progression free survival (PFS) in Chinese patients with metastatic renal cell carcinoma (mRCC).

**Methods:**

A multicenter, retrospective study was performed to elucidate the relationship between clinical variables and prognosis comparing sorafenib and sunitinib as first-line treatment agents in Chinese patients with mRCC. Between September 2006 and December 2014, 845 patients received either sorafenib (400 mg bid; *n* = 483) or sunitinib (50 mg q.d; *n* = 362). The primary end point was OS and PFS.

**Results:**

The percentage of patients with low and moderate risk according to Memorial Sloan-Kettering Cancer Centre (MSKCC) score was significantly higher in sunitinib group, and that with high risk was significantly higher in sorafenib group (15.1 vs. 5.2%; *p* < 0.001). Median OS was similar in sorafenib and sunitinib group (24 vs. 24 months; *p* = 0.298). Sorafenib group exhibited higher mPFS compared to sunitinib group (11.1 vs. 10.0 months; *p* = 0.028). Treatment (sorafenib vs sunitinib), pathology, Eastern Cooperative Oncology Group (ECOG) performance status, MSKCC scores, Heng’s criteria of risk, and number of metastases were identified as significant predictors for OS and along with liver metastasis for PFS. Clinical outcomes in terms of mOS was significantly better with sorafenib in patients ≥65 years of age (*p* = .041), ECOG 0 (*p* = 0.0001), and median MSKCC risk score (*p* = 0.008).

**Conclusions:**

Sorafenib and sunitinib are both effective in treating mRCC. However, sorafenib might be more effective in elderly patients (≥65 years) and in patients with an ECOG status of 0, classified under MSKCC moderate risk.

## Background

It is evident that ~30% of renal cell carcinoma (RCC) patients have overt metastases, defined as metastatic RCC (mRCC) [1] with a very poor average 5-year survival rate (only 10–12%) [2]. The growing evidence on the associations of molecular mechanisms with mRCC and also the abstinence of cytokine-based therapies due to high toxicity profile [3–5] has rationalized several randomized clinical trials on molecular targeted therapies such as sorafenib [6], bevacizumab [4], temsirolimus [7], sunitinib [8], pazopanib [9], everolimus [10], and axitinib [11] as first- and second-line treatment, which were found to be efficacious and safer than conventional immunotherapy. The availability of these targeted therapies has resulted in prolonged overall survival (OS) to approximately 2 years, thereby emerging as the standard of care in the management of mRCC. However, efficacy of drugs used in cancer chemotherapy is often associated with distinctive challenges due to infrequent occurrence of measurable disease, prolonged natural history of disease, diverse clinical characteristics and greater likelihood of contrary outcomes when treating elderly patients with more aggressive treatments [12, 13]. These challenges also influence regular, as well as accelerated regulatory approvals of drugs, which require extensive evidence of efficacy derived from clinical trials in addition to accommodating integral characteristics of disease and patient population [14]. Also, first-line therapies should be strategically chosen in order to devoid the need of sequential therapy with second-line therapies. For this purpose, comparing the efficacy of drugs may offer substantial evidence and guidance on the optimal use of targeted therapies. When evaluated as first-line treatment, axitinib demonstrated clinical efficacy and safety, but no significant progression free survival (PFS) benefit over sorafenib in a Phase III randomized comparison [15]. Further, sunitinib had similar efficacy as pazopanib in a non-inferiority trial [16]. Moreover, in case of sunitinib failure in advanced RCC, everolimus and axitinib appear to provide second-line PFS benefits [17]. On the other hand, recent Phase III Investigating Torisel as Second-Line Therapy (INTORSECT) trial reported no significant benefit of either temsirolimus or sorafenib as second-line treatment after sunitinib failure [7], though, temsirolimus demonstrated clinical efficacy as first-line therapy in poor risk patients [18]. Although sorafenib is a comparator agent in several clinical trials and often used as a second-line therapy, Chinese patients have been more responsive to sorafenib than western patients, hence, both sunitinib and sorafenib are widely recommended first-line therapies in China [19]. However, studies directly comparing efficacy of the two therapies in first-line settings which may guide the clinical decisions of mRCC treatment in Asian patients are limited. Although, a Korean study has reported comparable efficacy of the 2 drugs in mRCC patients, the findings were limited due to small patient population and a single centric retrospective design, warranting additional investigation [20]. Hence this study aims to retrospectively elucidate the relationship between clinical variables and disease prognosis by comparing sorafenib versus sunitinib in Chinese patients with mRCC at 3 tertiary hospitals in China.

## Methods

### Patient population

Records of patients with mRCC were maintained at the Beijing Cancer Hospital, Fudan University Shanghai Cancer Centre and the Peking University First Hospital. Between September 2006 and December 2014, the patient records were retrospectively reviewed and computed tomography (CT) scans were independently reviewed by a senior radiologist, blinded to a treatment arm.

Patients between 18 and 84 years of age; histological confirmation of advanced/mRCC; unsuitable for cytokine therapy; no prior systemic therapy; Eastern Cooperative Oncology Group performance status (ECOG PS) 0 to 3; 1 or more measurable lesions by CT or magnetic resonance imaging (MRI) according to Response Evaluation Criteria in Solid Tumors (RECIST 1.0); favorable or intermediate Memorial Sloan Kettering Cancer Centre (MSKCC) risk score; adequate bone marrow, liver, and renal function and willing to undergo first-line targeted therapy with sorafenib or sunitinib are included. Patients were excluded if they had unstable or severe cardiac disease; active, clinically serious infection or symptomatic metastatic brain tumor and with ECOG PS 4 and 5. Ethical approval was obtained from institutional ethics committee of Beijing Cancer Hospital, Fudan University Shanghai Cancer Centre and the Peking University First Hospital, and the protocol conformed to the principles of declaration of Helsinki, its subsequent revisions. Patient signed informed consent was obtained.

### Treatment

All the patients received first-line treatment with either sorafenib or sunitinib as monotherapy. Sorafenib was administered at a dose of 400 mg twice daily and sunitinib at a dose of 50 mg q.d. Dose reduction or temporary suspension was carried out if grade 3–4 adverse event (AE) was reported according to the local prescribing information (PI). However, sorafenib dosage was increased to 600 mg twice daily and subsequently to 800 mg twice daily in some patients with disease progression.

### Outcomes and assessments

The primary endpoint was OS (calculated from the date of first dose of sorafenib to the date of death or last follow-up) and PFS (time from first administration of sorafenib to the first documentation of disease progression or death from any cause). The effect of important prognostic factors such as age, gender, MSKCC score, ECOG performance and number of metastatic tumors on PFS and OS were evaluated.

### Statistical analysis

Continuous variables such as PFS and OS were reported as medians and interquartile ranges, and categorical data such as age, gender, previous nephrectomy or systemic therapy were presented as proportions. The follow-up duration was calculated using reversed Kaplan-Meier method. The Shapiro-Wilk test was used to evaluate the data for normality distribution. OS and PFS were estimated using the Kaplan-Meier method with Rothman’s 95% CI and compared across groups using the log-rank test. The Cox proportional hazards model was used to evaluate the prognostic value of investigated parameters. All *p* values were two-sided and were considered significant if <.05. The concordance index and the proportion of 2 explained variance (R) was computed to assess the prediction performance for survival (PFS and OS). The statistical analysis of the collected data was performed using SPSS software version 19.

## Results

### Patients and baseline demographics

The data of 845 patients with mRCC enrolled between September 2006 and December 2014 were analyzed. Baseline demographics and clinical characteristics of the study population are presented in Table 1. Of the 845 patients, 483 were treated with sorafenib and 362 were treated with sunitinib. Majority of patients were ≤65 years of age and were predominantly men in both the treatment groups (age ≤65 years: 77.2% vs. 85.4%; males: 73.1% vs. 76.2% in sorafenib and sunitinib group, respectively). Approximately, 48% of the patients in the sorafenib group and 66% in the Sunitinib group had an ECOG performance status of 0, and majority of patients in both the groups were at moderate risk according to the MSKCC score (49.1% vs. 53%) and Heng’s score (47% vs 51.7%). There were significantly more number of patients with non-clear cell-type RCC in the sorafenib group (15.5% vs. 8.6%; *p* = 0.002). However, the number of patients at low and moderate risk according to MSKCC score were significantly more in the sunitinib group and the number of patients at high risk according to MSKCC score were significantly more in the sorafenib group (15.1% vs. 5.2%; *p* < 0.001). No significant differences between the 2 treatment groups were observed for parameters such as gender, number of metastases, bone metastasis and simple bone metastasis.

**Table 1:** Baseline characteristics of the study population

Clinical variable		Sorafenib, n (%)	Sunitinib, n (%)	*P*
Age (years)	<65	373 (77.2)	309 (85.4)	0.004
	≥65	110 (22.8)	53 (14.6)	
Gender	Male	353 (73.1)	276 (76.2)	0.302
	Female	130 (26.9)	86 (23.8)	
Pathology	Clear cell type	408 (84.5)	331 (91.4)	0.002
	Non-clear cell type	75 (15.5)	31 (8.6)	
ECOG	0	230 (47.6)	238 (65.7)	<0.001
	1	188 (38.9)	94 (26.0)	
	2	58 (12.0)	29 (8.0)	
	3	7 (1.4)	1 (0.3)	
Previous nephrectomy	Yes	376 (77.8)	298 (82.3)	0.120
	No	107 (22.2)	64 (17.7)	
MSKCC	Low risk	173 (35.8)	151 (41.7)	<0.001
	Moderate risk	237 (49.1)	192 (53.0)	
	High risk	73 (15.1)	19 (5.2)	
Heng’s criteria	Low risk	183 (37.9)	144 (39.8)	0.016
	Moderate risk	227 (47.0)	187 (51.7)	
	High risk	73 (15.1)	31 (8.6)	
Number of metastatic organs	1	207 (42.9)	174 (48.1)	0.129
	2	182 (37.7)	109 (30.1)	
	3	75 (15.5)	66 (18.2)	
	4	19 (3.9)	13 (3.6)	
Lung metastasis	No	174 (36.0)	106 (29.3)	0.046
	Yes	309 (64.0)	256 (70.7)	
Simple lung metastasis	No	380 (78.7)	251 (69.3)	0.002
	Yes	103 (21.3)	111 (30.7)	
Bone metastasis	No	319 (66.0)	254 (70.2)	0.207
	Yes	164 (34.0)	108 (29.8)	
Simple bone metastasis	No	445 (92.1)	341 (94.2)	0.276
	Yes	38 (7.9)	21 (5.8)	
Liver metastasis	No	421 (87.2)	335 (92.5)	0.013
	Yes	62 (12.8)	27 (7.5)	
Lymph node metastasis	No	323 (66.9)	248 (68.5)	0.656
	Yes	260 (33.1)	114 (31.5)	
RECIST response	CR	5 (1.0)	4 (1.1)	
	PR	77 (15.9)	72 (19.9)	
	SD	346 (71.6)	244 (67.4)	
	PD	55 (11.4)	42 (11.6)	0.110
Second line treatment	Yes	132 (27.3)	81 (22.4)	
	No	351 (72.7)	281 (77.6)	

*ECOG* eastern cooperative oncology group, *MSKCC* memorial sloan-kettering cancer centre, *OS* overall survival, *PFS* progression-free survival

### Endpoint analysis

**Fig. 1:**
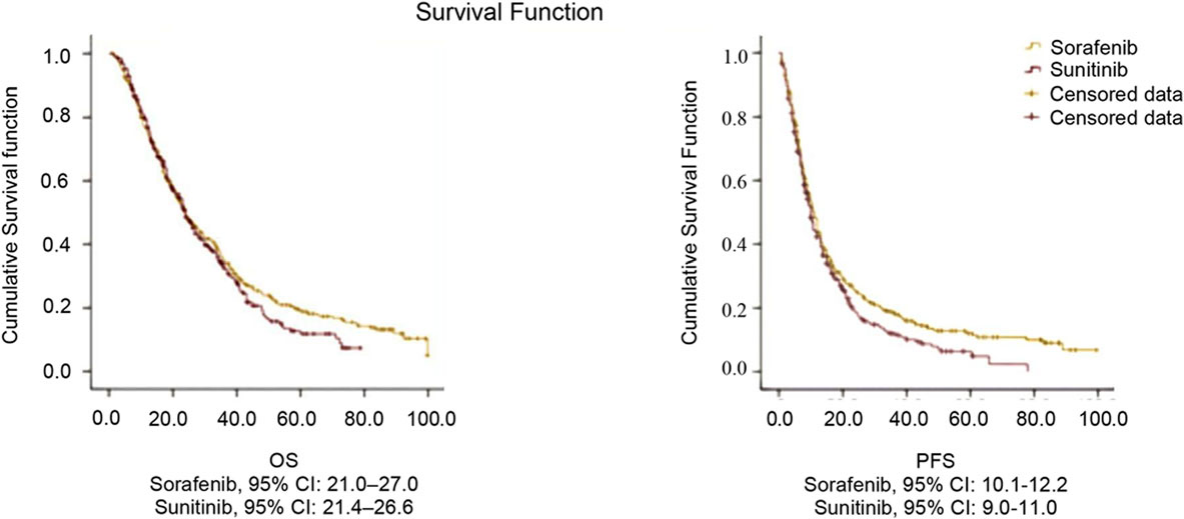
Survival data of first-line targeted therapy for advanced renal carcinoma

Survival data are presented in terms of OS and PFS. Median OS (mOS) and PFS (mPFS) for both the treatment groups are shown in Fig. 1. Median OS (months) for the sorafenib and sunitinib groups was found to be similar (24.0 vs. 24.0; *p* = 0.298). Overall, the sorafenib group exhibited higher mPFS (months) when compared to sunitinib group (11.1 vs. 10.0; *p* = 0.028). Overall response rate (CR + PR) of sorafenib treatment was 16.77% (82/483) was lower than sunitinib treatment 20.99% (76/362). Disease control rate (CR + PR + SD) was similar in 2 groups 88.61% (428/483) vs 88.39% (320/362). Disease progression was seen in 11.4% patients in sorafenib group and 11.6% in sunitinib. Only 27.3% in the sorafenib group and 22.4% in the sunitinib group had dose escalation which was considered as second line treatment.

### Predictor analysis

**Fig. 2:**
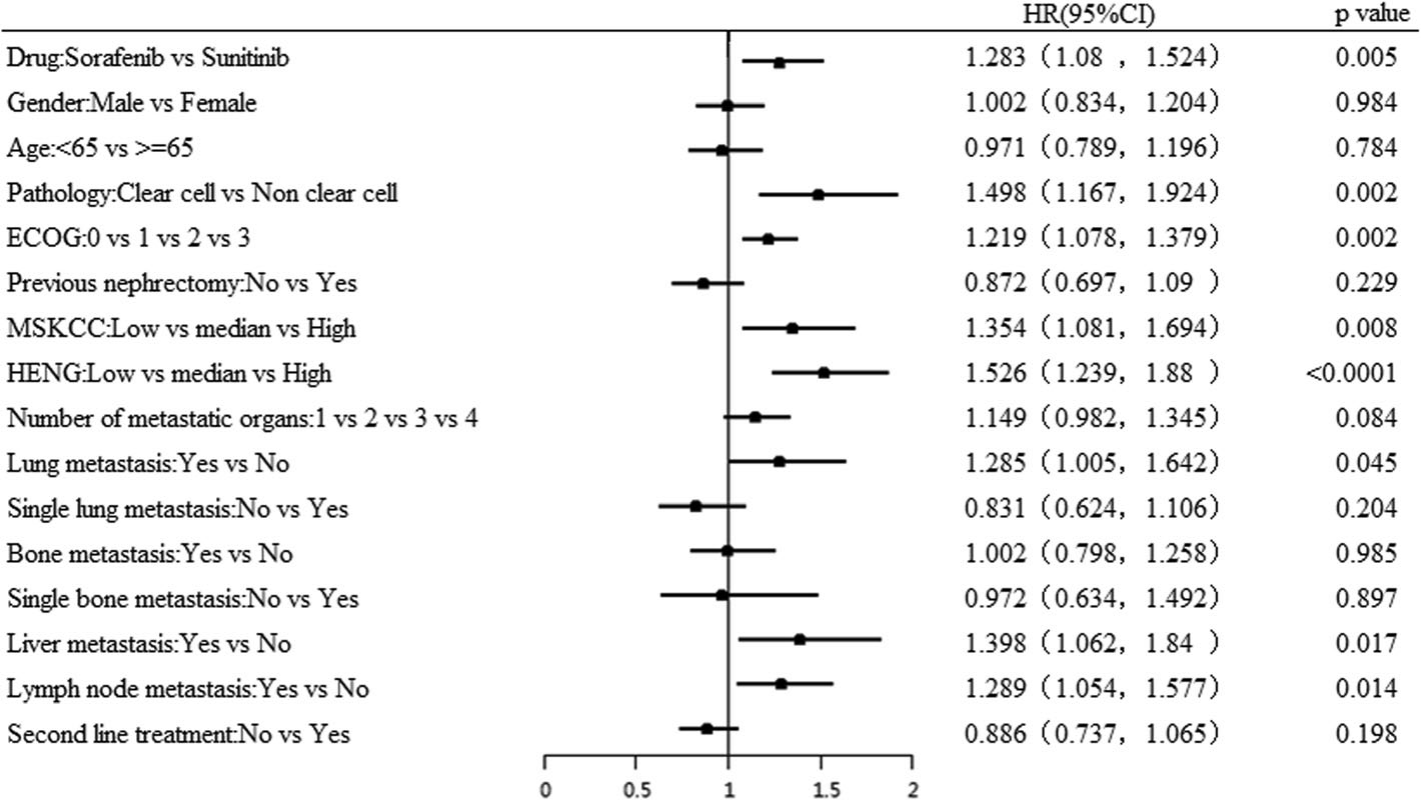
Multivariate analysis of predictors of OS. ECOG, Eastern Cooperative Oncology Group; MSKCC, Memorial Sloan-Kettering Cancer Centre; OS, overall survival

**Fig. 3:**
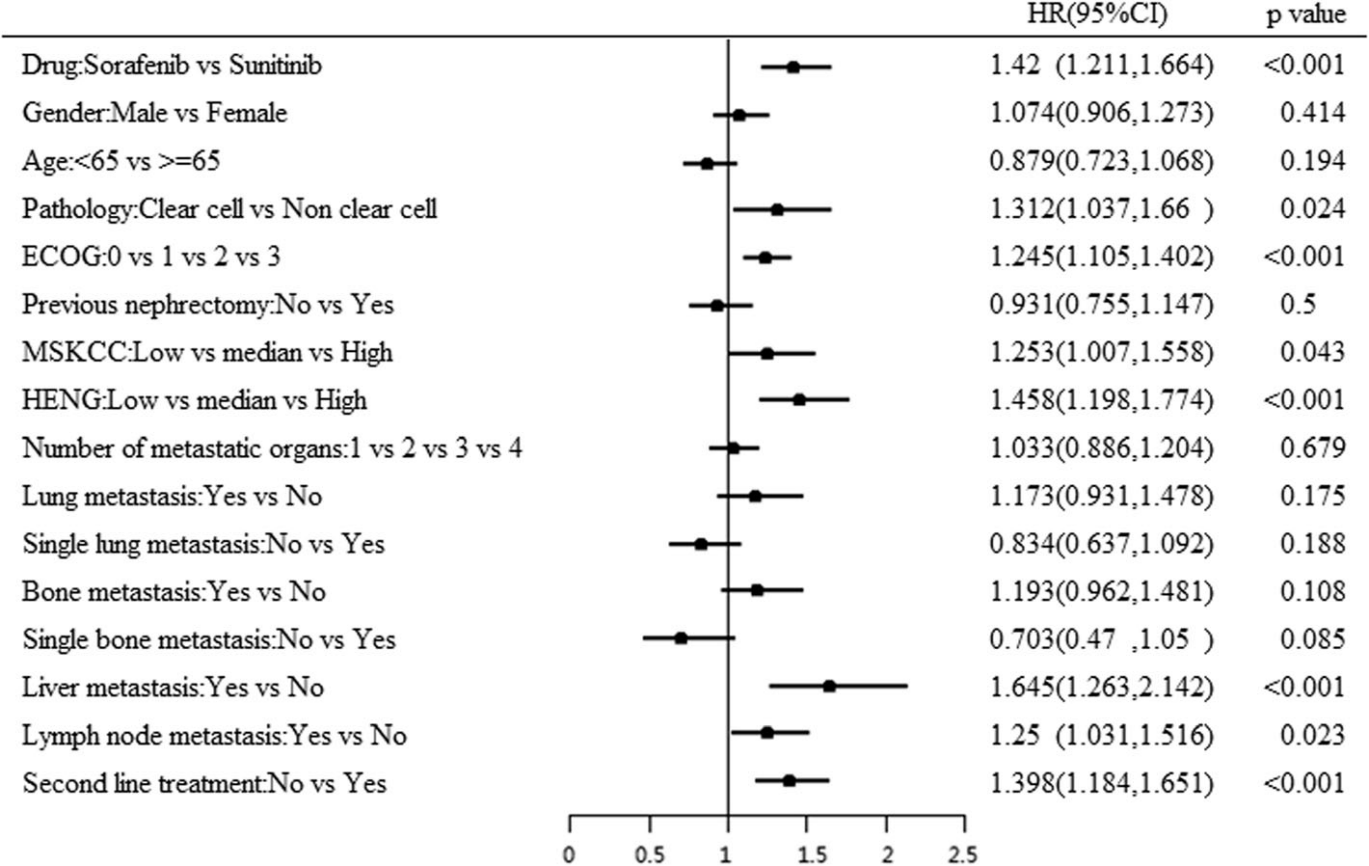
Multivariate analysis of predictors of PFS. ECOG, Eastern Cooperative Oncology Group; MSKCC, Memorial Sloan-Kettering Cancer Centre; OS, overall survival

Univariate analysis of 12 key including demographic and clinical characteristics identified pathology of RCC (clear cell and non-clear cell type), ECOG performance status, MSKCC score for risk, Heng’s criteria for risk, number of metastasis, simple lung metastasis, bone metastasis and liver metastasis as significant predictors for OS (*p* = 0.000) and PFS (*p* = 0.000). Additionally, simple bone metastasis was also identified as a significant predictor of PFS (*p* = 0.016). Data from univariate analysis of OS and PFS predictors are detailed in Table 2. However, multivariate analysis identified variables such as treatment (sorafenib vs. sunitinib), pathology, ECOG performance status, MSKCC scores, Heng’s criteria of risk and number of metastases as significant predictors for OS (Fig. 2). For PFS, liver metastasis along with other variables reported for OS were identified as significant predictors (Table 3, Fig. 3). Clinical outcomes in terms of mOS seemed to be significantly better with sorafenib in patients older than 65 years (*p* = 0.041), ECOG of 0 (*p* < 0.001) and median MSKCC risk score (*p* = 0.008).

**Table 2:** Univariate analysis of predictors for OS and PFS

Clinical variable		mOS (months)	Log Rank test, *P*	mPFS (months)	Log Rank test, *P*
Gender	Male	24.0	0.413	11.0	0.131
	Female	23.0		10.0	
Age	<65 years	24.0	0.714	10.6	0.435
	≥65 years	24.0		11.0	
Pathology	Clear cell type	25.5	<0.001	11.3	<0.001
	Non-clear cell type	14.0		7.0	
ECOG	0	30.0	<0.001	12.1	<0.001
	1	22.1		10.6	
	2	11.0		5.9	
	3	8.8		3.4	
Previous nephrectomy	Yes	26.7	<0.001	11.6	<0.001
	No	14.0		7.0	
MSKCC	Low risk	39.0	<0.001	15.0	<0.001
	Moderate risk	22.0		9.5	
	High risk	9.3		5.2	
HENG	Low risk	39.0	<0.001	15.0	<0.001
	Moderate risk	22.0		9.4	
	High risk	10.3		5.8	
Number of metastatic organs	1	32.0	<0.001	14.0	<0.001
	2	21.0		9.6	
	3	15.3		8.0	
	4	16.0		7.0	
Lung metastasis	No	23.0	0.362	10.0	0.429
	Yes	24.0		11.0	
Simple lung metastasis	No	21.4	<0.001	9.3	<0.001
	Yes	32.4		15.0	
Bone metastasis	No	26.0	<0.001	12.0	0.001
	Yes	20.4		9.0	
Simple bone metastasis	No	24.0	0.182	10.5	0.016
	Yes	24.0		12.0	
Liver metastasis	No	25.0	<0.001	11.3	<0.001
	Yes	15.0		6.0	
Lymph node metastasis	No	27.5	<0.001	12.0	<0.001
	Yes	18.0		9.0	
RECIST response	CR	60.0	<0.001	31.5	<0.001
	PR	36.0		20.7	
	SD	23.3		10.4	
	PD	8.3		3.0	
Second line treatment	Yes	30.0	0.024	10.9	0.363
	No	22.0		10.5	

*ECOG* eastern cooperative oncology group, *MSKCC* memorial sloan-kettering cancer centre, *OS* overall survival, *PFS* progression-free survival

**Table 3:** Multivariate analysis of predictors for PFS

Variables		*P*	HR	95.0% CI	
				Lower	Upper
Drug	Sorafenib vs Sunitinib	<0.001	1.420	1.211	1.664
Gender	Male vs Female	0.414	1.074	0.906	1.273
Age	<65 vs > =65	0.194	0.879	0.723	1.068
Pathology	Clear cell vs Non clear cell	0.024	1.312	1.037	1.660
ECOG	0 vs 1 vs 2 vs 3	<0.001	1.245	1.105	1.402
Previous nephrectomy	No vs Yes	0.500	0.931	0.755	1.147
MSKCC	Low vs median vs High	0.043	1.253	1.007	1.558
HENG	Low vs median vs High	<0.001	1.458	1.198	1.774
Number of metastatic organs	1 vs 2 vs 3 vs 4	0.679	1.033	0.886	1.204
Lung metastasis	Yes vs No	0.175	1.173	0.931	1.478
Single lung metastasis	No vs Yes	0.188	0.834	0.637	1.092
Bone metastasis	Yes vs No	0.108	1.193	0.962	1.481
Single bone metastasis	No vs Yes	0.085	0.703	0.470	1.050
Liver metastasis	Yes vs No	<0.001	1.645	1.263	2.142
Lymph node metastasis	Yes vs No	0.023	1.250	1.031	1.516
Second line treatment	No vs Yes	<0.001	1.398	1.184	1.651

Further, multivariate analysis revealed significant association between OS and several predictors such as sorafenib treatment (HR 1.3, 95% CI 1.096, 1.542; *p* = 0.003), Clear cell type RCC (HR 1.49, 95% CI 1.167, 1.922; *p* = 0.002), ECOG grade 3 (HR 1.22, 95% CI 1.082, 1.385; *p* = 0.001), high grade MSKCC 3 (HR 1.36, 95% CI 1.086, 1.703; *p* = 0.007), Heng risk (HR 1.53, 95% CI 1.244, 1.889; *p* < 0.0001), presence of liver (HR 1.40, 95% CI 1.067, 1.846; *p* = 0.001), and lymph node metastases (HR 1.22, 95% CI 1.082, 1.385; *p* = 0.001). Other prognostic factors such as gender, age, second line treatment, lung and bone metastasis and previous nephrectomy showed no significant association with OS (Table 4).

**Table 4:** Multivariate analysis of predictors for OS

Variables		*P*	HR	95.0% CI	
				Lower	Upper
Drug	Sorafenib vs Sunitinib	0.005	1.283	1.080	1.524
Gender	Male vs Female	0.984	1.002	0.834	1.204
Age	<65 vs > =65	0.784	0.971	0.789	1.196
Pathology	Clear cell vs Non clear cell	0.002	1.498	1.167	1.924
ECOG	0 vs 1 vs 2 vs 3	0.002	1.219	1.078	1.379
Previous nephrectomy	No vs Yes	0.229	0.872	0.697	1.090
MSKCC	Low vs median vs High	0.008	1.354	1.081	1.694
HENG	Low vs median vs High	<0.0001	1.526	1.239	1.880
Number of metastatic organs	1 vs 2 vs 3 vs 4	0.084	1.149	0.982	1.345
Lung metastasis	Yes vs No	0.045	1.285	1.005	1.642
Single lung metastasis	No vs Yes	0.204	0.831	0.624	1.106
Bone metastasis	Yes vs No	0.985	1.002	0.798	1.258
Single bone metastasis	No vs Yes	0.897	0.972	0.634	1.492
Liver metastasis	Yes vs No	0.017	1.398	1.062	1.840
Lymph node metastasis	Yes vs No	0.014	1.289	1.054	1.577
Second line treatment	No vs Yes	0.198	0.886	0.737	1.065

## Discussion

Sorafenib and sunitinib have been used for mRCC in China since 2007. Since then several studies have tried to elucidate the efficacy of the novel treatments. OS is a reliable endpoint for assessing the efficacy of mRCC with targeted therapy [21]. Although, these new therapies have improved the OS and PFS of patients with mRCC, gradual development of drug resistance may often lead to switching one therapy to other, resulting in sequential therapy. Filson et al. reported that patients on first-line sorafenib therapy have high probability of proceeding to second-line therapy with sunitinib [22]. This clearly indicates the difference between the efficacies of the two drugs. However, a Korean study revealed comparable efficacy outcomes of sorafenib and sunitinib when used as monotherapy in first-line settings [20]. In support to the Korean study, more Asian studies are warranted to elucidate the efficacies of these therapies to inform the choice of first-line therapies.

Evidence-based studies suggest both first- and second-line efficacy of sorafenib. Earlier trials have shown comparable efficacy of sorafenib with interferon alpha-2a (PFS: 5.7 vs 5.6 months respectively), were the sorafenib treatment was well tolerated by the patients [23]. Also, in the INTORSECT trial, sorafenib had significantly longer OS compared to temsirolimus in first line therapy [7]. Further, a non-randomized open access trial demonstrated that sorafenib provides similar benefits in both first- and second-line setting [15]. The recent findings from an Italian study further confirmed that sorafenib prolonged PFS and OS in both first- and second-line routine community practice setting [24]. On the other hand, sunitinib also has established efficacy in previous phase 3 trials. Further, the efficacy of sunitinib was found to be superior to IFN-alpha but comparable to pazopanib across the trails, however severe grade 3 or 4 adverse effects were the limitations [8, 25].

Though efficacies of both the drugs in first-line setting are well described in retrospective literature, head-to-head comparison of real world clinical outcomes in patients are more heterogeneous than those trailed under controlled conditions, were patients with independent prognostic risk factors such as elderly, ECOG performance status and MSKCC Moderate risk groups were excluded. Considering the limitation of the clinical trials, expanded-access studies were conducted in America [26] and Europe [27] on sorafenib and one study on sunitinib [28], which were close to the real world scenario. Further, patient age may be a pivotal prognostic factor as elderly patients tend to have lower ECOG performance status than younger patients [29]. Hence, head-to-head comparisons are needed to inform the choice of treatment for such selected patients.

Our previous report documented higher clinical benefit rate in sorafenib treated patients than sunitinib treated patients (94.67% vs 84.33%) [30]. In extension to this, the present retrospective review reported similar effectiveness of sorafenib and sunitinib in treating Chinese patients with mRCC, however, sorafenib therapy was more effective in elderly patients (≥65 years) and in patients with an normal performance status who were classified under MSKCC moderate risk category. A sub analysis of elderly patients in a phase 3 trial revealed a significant PFS advantage of sorafenib regardless of age [19]. In contrast, expanded-access studies on the clinical outcomes of sunitinib reproduced consistent efficacy and safety outcomes with previous results, and the outcomes were fairly similar in both elderly and younger populations but with significantly more common adverse effects seen in older population. However the clinical benefits of OS and PFS were inferior compared with placebo [28]. In line with our findings, a Swedish register-based demonstrated no difference between sorafenib and sunitinib in the duration of treatment or time to death when used as first-line therapy, however, the impact of the duration of first-line treatment differed in sequential therapy, concluding sorafenib first line treatment as a favorable choice in mRCC [31]. Furthermore, comparison of the present mOS findings with sorafenib therapy with other Asian studies demonstrated mixed results, where the mOS was consistent with a Chinese study12 conducted by Yu et al. [32], but were lower than the other Chinese [33, 34] and Korean studies [20]. However, higher mPFS with sorafenib over sunitinib was demonstrated in this study compared to previous Chinese [32], Korean [20, 35] and Italian [24] studies. The discrepancy may be related to diversity of patient populations enrolled in each study differing in many aspects related to prognosis and ethnicity. Furthermore, studies suggest that compared with Western patients, Chinese patients respond better to sorafenib as first-line targeted therapy [36, 37] Also, the results from TIVO-1 trial suggested that sorafenib as a first-line mRCC therapy yielded PFS of 9.1 month [38], which was lesser than the findings from the present study (PFS 11.1 months). Hence, the present study findings further support the previous findings with regards to superior efficacy of sorafenib in Chinese patients with mRCC.

Furthermore, multivariate regression analysis revealed treatment (Sorafenib vs. Sunitinib), pathology, ECOG performance status, MSKCC scores, HENG criteria of risk, and number of metastases as significant predictors for OS which is in line with the previous studies conducted by Motzar et al., which demonstrated ethnicity, ECOG status, bone metastasis and old age as predictors for OS and PFS in first-line therapy with sunitinib [39]. The findings are also consistent with the findings of Yang et al. who reported MSKCC status as prognostic factor for OS and PFS when treated with sunitinib [40]. Overall, our study findings support the findings of the Swedish and Italian studies, demonstrating comparable efficacy of sorafenib over sunitinib, but a more favorable sorafenib therapy in elderly and moderate risk mRCC patients.

Our study has several limitations. The retrospective design of the study comes as an inherent limitation; however, relatively large sample size compared to previous retrospective studies may power the study. The favorable outcomes of sorafenib demonstrated in this study may create a conflict in the belief of the clinicians who believe sunitinib as a better first-line option. Further, mOS was chosen as an endpoint and hence, the survival probability at a later time point after treatment initiation could not be established, hence, warranting further long term comparative efficacy studies.

## Conclusion

The present study suggests that sorafenib and sunitinib are both effective as first-line therapeutic agents in treating Chinese patients with mRCC. Sorafenib is effective in elderly patients (≥65 years) and in patients with an ECOG status of 0, classified under MSKCC moderate risk. In addition, multivariate analysis suggests that variables such as treatment (sorafenib vs sunitinib), pathology, ECOG performance status, MSKCC scores, Heng’s criteria of risk and number of metastases are significant prognostic factors for OS and PFS.

## Abbreviations

AE: Adverse effect
CT: Computer tomography
DFS: Disease free survival
ECOCG: Eastern cooperative oncology group performance status
mOS: Median overall survival
mPFS: Median progression free survival
mRCC: Metastatic renal cell carcinoma
MRI: Magnetic resonance imaging
MSKCC: Memorial sloan-kettering cancer centre
ORR: Objective response rate
OS: Overall survival
PFS: Progression free survival
PI: Prescribing information
QoL: Quality of life
RECIST: Response evaluation criteria in solid tumors
